# 4-[(4-Methyl­benz­yl)amino]-3-[(4-methyl­benz­yl)imino­meth­yl]-2*H*-chromen-2-one

**DOI:** 10.1107/S1600536810041218

**Published:** 2010-10-20

**Authors:** D. Rambabu, G. Rama Krishna, C. Malla Reddy, Manojit Pal

**Affiliations:** a Institute of Life Sciences, University of Hyderabad Campus, Hyderabad 500 046, India; b Department of Chemical Sciences, Indian Institute of Science Education and Research, Kolkata, West Bengal, 741 252, India

## Abstract

The title compound, C_26_H_24_N_2_O_2_, was prepared from the reaction of 4-chloro-3-formyl­coumarin with *p*-methyl­benzyl­amine. Even though there are no strong and specific inter­actions in the crystal structure, the translationally related mol­ecules form chains along the *b* axis. The coumarin moieties are stacked through π–π inter­actions [centroid–centroid distance = 3.5275 (7) Å], forming layers perpendicular to the stacking direction.

## Related literature

For the medicinal and biological activity of coumarins and their derivatives, see: Borges *et al.* (2005 ▶); Kontogiorgis & Hadjipavlou-Litina (2005 ▶); Gürsoy & Karali (2003 ▶); Pratibha & Shreeya (1999 ▶); Manolov & Danchev (1995 ▶). 
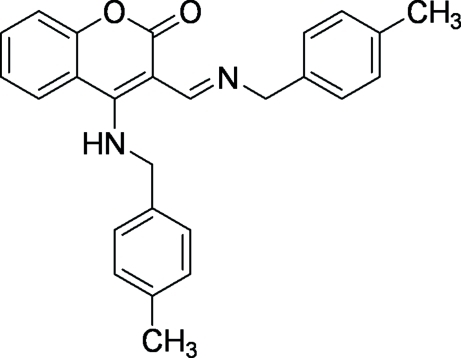


## Experimental

### 

#### Crystal data

C_26_H_24_N_2_O_2_
*M* *_r_* = 396.47
Triclinic, 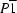

*a* = 6.8137 (2) Å
*b* = 9.2636 (3) Å
*c* = 17.4364 (5) Å
α = 99.525 (2)°
β = 97.423 (2)°
γ = 107.251 (2)°
*V* = 1017.84 (6) Å^3^
*Z* = 2
Mo *K*α radiation
μ = 0.08 mm^−1^
*T* = 296 K
0.32 × 0.26 × 0.21 mm

#### Data collection

Bruker Kappa APEXII CCD DUO diffractometer
17607 measured reflections
4391 independent reflections
3867 reflections with *I* > 2σ(*I*)
*R*_int_ = 0.027

#### Refinement

*R*[*F* ^2^ > 2σ(*F* ^2^)] = 0.039
*wR*(*F* ^2^) = 0.122
*S* = 1.08
4391 reflections
277 parameters
H atoms treated by a mixture of independent and constrained refinement
Δρ_max_ = 0.36 e Å^−3^
Δρ_min_ = −0.37 e Å^−3^

### 

Data collection: *APEX2* (Bruker, 2008 ▶); cell refinement: *SAINT* (Bruker, 2008 ▶); data reduction: *SAINT*; program(s) used to solve structure: *SHELXS97* (Sheldrick, 2008 ▶); program(s) used to refine structure: *SHELXL97* (Sheldrick, 2008 ▶); molecular graphics: *X-SEED* (Barbour, 2001 ▶); software used to prepare material for publication: *SHELXL97*.

## Supplementary Material

Crystal structure: contains datablocks I, global. DOI: 10.1107/S1600536810041218/bh2311sup1.cif

Structure factors: contains datablocks I. DOI: 10.1107/S1600536810041218/bh2311Isup2.hkl

Additional supplementary materials:  crystallographic information; 3D view; checkCIF report

## Figures and Tables

**Fig. 1.**
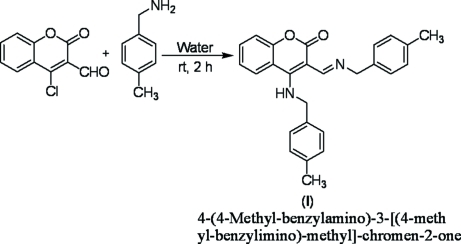
Synthetic route for the title compound (I)

**Fig. 2.**
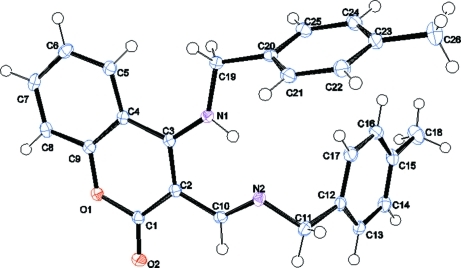
ORTEP representation of (I), with displacement ellipsoids drawn at the 50% probability level.

**Fig. 3.**
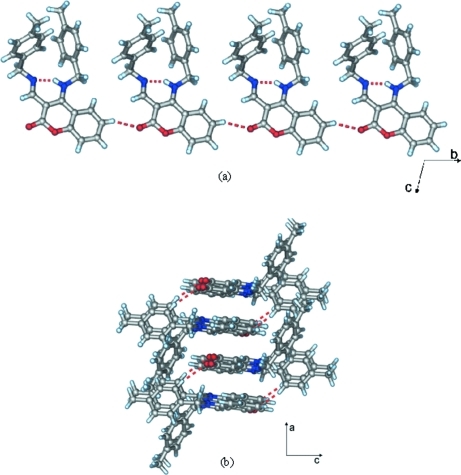
Crystal packing of (I): (a) showing the one dimensional chain formed via weak C—H···O hydrogen bonds along the b-axis. (intramolecular N—H···N interaction can also be seen). (b) Coumarin π-stacked layers along the a axis and stabilized by weak C—H···O interactions.

## supplementary crystallographic information

### Comment

Coumarin is an important structural framework present in a variety of natural and synthetic products that possess significant biological activity (Borges *et al.*, 2005; Gürsoy & Karali, 2003; Kontogiorgis & Hadjipavlou-Litina, 2005). Coumarin derivatives have been shown to possess a remarkably broad spectrum of biological activity, including anti-inflammatory, antibacterial (Pratibha & Shreeya, 1999) anticancer, antiviral, antitumor, anticoagulant, antifungal (Manolov & Danchev, 1995) and anti-HIV activity. During our attempts to synthesize benzazepine derivatives containing various substituents on the benzene ring, the title compound, (I), was obtained unexpectedly by the formation of coumarin derivative instead of the benzazepine derivative (Fig. 1).

The compound (I) was crystallized in the triclinic, *P*-1 space group with one molecule in the asymmetric unit (*Z*'=1) (Fig. 2). The crystal structure analysis reveals that the coumarin moieties in the crystal form layers with weak *π*···*π* interactions. The molecular structure shows that the 4-methylbenzyl amine and imine moieties form an intramolecular N—H···N interaction [*D* = N···N = 2.6233 (15) Å, *θ* = 147.7 (18)°]. The torsion angle of amine attached 4-methylbenzyl group (C24—C25—C20—C19) is 179.09° and imine attached 4-methylbenzyl group (C16—C17—C12—C11) is 172.16°. There are no strong and specific intermolecular interactions found in the crystal structure. The two conformationally flexible moieties (4-methylbenzyl groups attached to amine and imine groups) are however stabilized by C—H···*π* interactions [C17—H17···*π*(C20···C25): 2.667 Å, 154.61°]. The translational related molecules interact with each other *via* weak C—H···O [C6—H6···O2: H6···O2 = 2.615 Å, *θ* = 134.49°] hydrogen bonds along the *b*-axis, and form a one dimensional chain (Fig. 3*a*). The inversion related molecules form weak coumarin *π*–stacked layers and these layers are stabilized by weak C—H···O [C21—H21···O1: *d* = H21···O1 = 2.620 Å, *θ* = 159.37°] hydrogen bonds (Fig.3*b*).

### Experimental

A mixture of 4-chloro-3-formylcoumarin (1.0 mmol) and *p*-methylbenzylamine (2.0 mmol) were stirred in water (15 ml) at room temperature for 2 h (Fig. 1). After completion of the reaction, the solid product was filtered. The crude product was crystallized from DMF and (I) was obtained as colourless needles by slow evaporation.

### Refinement

The crystal structure was solved by direct methods using *SHELXS97* and refined by full matrix least-squares refinement on *F*^2^ with anisotropic displacement parameters for non-H atoms, using *SHELXL97*. NH hydrogen atom (H1) was located in a difference map and refined freely. Aromatic and aliphatic CH hydrogen atoms were generated by the riding model in idealized geometries.

### Crystal data

**Table:**

C_26_H_24_N_2_O_2_	*Z* = 2
*M**_r_* = 396.47	*F*(000) = 420.0
Triclinic, *P*1	*D*_x_ = 1.294 Mg m^−^^3^
Hall symbol: -P 1	Mo *K*α radiation, λ = 0.71073 Å
*a* = 6.8137 (2) Å	Cell parameters from 213 reflections
*b* = 9.2636 (3) Å	θ = 2.4–27.0°
*c* = 17.4364 (5) Å	µ = 0.08 mm^−^^1^
α = 99.525 (2)°	*T* = 296 K
β = 97.423 (2)°	Needle, colourless
γ = 107.251 (2)°	0.32 × 0.26 × 0.21 mm
*V* = 1017.84 (6) Å^3^	

### Data collection

**Table:**

Bruker Kappa APEXII CCD DUO diffractometer	3867 reflections with *I* > 2σ(*I*)
Radiation source: fine-focus sealed tube	*R*_int_ = 0.027
graphite	θ_max_ = 27.0°, θ_min_ = 2.4°
φ and ω scans	*h* = −7→8
17607 measured reflections	*k* = −11→11
4391 independent reflections	*l* = −21→22

### Refinement

**Table:**

Refinement on *F*^2^	Primary atom site location: structure-invariant direct methods
Least-squares matrix: full	Secondary atom site location: difference Fourier map
*R*[*F*^2^ > 2σ(*F*^2^)] = 0.039	Hydrogen site location: inferred from neighbouring sites
*wR*(*F*^2^) = 0.122	H atoms treated by a mixture of independent and constrained refinement
*S* = 1.08	*w* = 1/[σ^2^(*F*_o_^2^) + (0.063*P*)^2^ + 0.4639*P*] where *P* = (*F*_o_^2^ + 2*F*_c_^2^)/3
4391 reflections	(Δ/σ)_max_ = 0.015
277 parameters	Δρ_max_ = 0.36 e Å^−^^3^
0 restraints	Δρ_min_ = −0.37 e Å^−^^3^
0 constraints	

### Fractional atomic coordinates and isotropic or equivalent isotropic displacement parameters (Å^2^)

**Table:**

	*x*	*y*	*z*	*U*_iso_*/*U*_eq_	
O1	0.71493 (13)	0.94920 (10)	1.10649 (5)	0.0147 (2)	
O2	0.68675 (15)	0.70231 (11)	1.08127 (5)	0.0202 (2)	
N1	0.76368 (16)	0.96900 (12)	0.87229 (6)	0.0135 (2)	
N2	0.70561 (15)	0.67083 (12)	0.84586 (6)	0.0140 (2)	
C1	0.70620 (18)	0.81679 (14)	1.05335 (7)	0.0135 (2)	
C2	0.72049 (17)	0.82812 (14)	0.97287 (7)	0.0121 (2)	
C3	0.75128 (17)	0.97000 (14)	0.94846 (7)	0.0114 (2)	
C4	0.76930 (17)	1.10806 (14)	1.00772 (7)	0.0121 (2)	
C5	0.80667 (18)	1.25968 (14)	0.99476 (8)	0.0149 (3)	
H5	0.8216	1.2774	0.9445	0.018*	
C6	0.82167 (19)	1.38246 (14)	1.05493 (8)	0.0165 (3)	
H6	0.8453	1.4810	1.0447	0.020*	
C7	0.80156 (18)	1.35920 (15)	1.13092 (8)	0.0168 (3)	
H7	0.8122	1.4420	1.1713	0.020*	
C8	0.76572 (19)	1.21248 (15)	1.14600 (7)	0.0160 (3)	
H8	0.7518	1.1961	1.1965	0.019*	
C9	0.75060 (17)	1.08969 (14)	1.08509 (7)	0.0132 (2)	
C10	0.69080 (18)	0.68181 (14)	0.91907 (7)	0.0128 (2)	
H10	0.6596	0.5920	0.9388	0.015*	
C11	0.6554 (2)	0.51577 (14)	0.79593 (8)	0.0169 (3)	
H11A	0.6342	0.4390	0.8284	0.020*	
H11B	0.7703	0.5106	0.7693	0.020*	
C12	0.45765 (19)	0.48263 (14)	0.73518 (7)	0.0150 (3)	
C13	0.2852 (2)	0.35067 (14)	0.72762 (7)	0.0173 (3)	
H13	0.2958	0.2758	0.7560	0.021*	
C14	0.0973 (2)	0.33028 (15)	0.67797 (8)	0.0190 (3)	
H14	−0.0155	0.2411	0.6732	0.023*	
C15	0.0748 (2)	0.44085 (15)	0.63519 (8)	0.0190 (3)	
C16	0.2498 (2)	0.57018 (15)	0.64110 (8)	0.0195 (3)	
H16	0.2401	0.6441	0.6120	0.023*	
C17	0.4385 (2)	0.59027 (15)	0.68983 (8)	0.0177 (3)	
H17	0.5536	0.6766	0.6922	0.021*	
C18	−0.1334 (2)	0.42037 (19)	0.58467 (9)	0.0285 (3)	
H18A	−0.1754	0.3258	0.5453	0.043*	
H18B	−0.1202	0.5060	0.5591	0.043*	
H18C	−0.2368	0.4163	0.6175	0.043*	
C19	0.76994 (19)	1.08754 (14)	0.82548 (7)	0.0147 (2)	
H19A	0.8992	1.1736	0.8443	0.018*	
H19B	0.6536	1.1262	0.8306	0.018*	
C20	0.75631 (19)	1.01513 (14)	0.73987 (7)	0.0145 (3)	
C21	0.9152 (2)	0.95875 (15)	0.71825 (8)	0.0168 (3)	
H21	1.0271	0.9651	0.7568	0.020*	
C22	0.9072 (2)	0.89349 (15)	0.63983 (8)	0.0198 (3)	
H22	1.0138	0.8563	0.6266	0.024*	
C23	0.7413 (2)	0.88269 (15)	0.58023 (8)	0.0200 (3)	
C24	0.5838 (2)	0.93895 (15)	0.60216 (8)	0.0198 (3)	
H24	0.4720	0.9328	0.5636	0.024*	
C25	0.5906 (2)	1.00432 (15)	0.68088 (8)	0.0176 (3)	
H25	0.4836	1.0410	0.6941	0.021*	
C26	0.7341 (3)	0.81009 (19)	0.49520 (9)	0.0309 (3)	
H26A	0.6987	0.7000	0.4891	0.046*	
H26B	0.8686	0.8511	0.4815	0.046*	
H26C	0.6305	0.8331	0.4610	0.046*	
H1	0.751 (3)	0.876 (2)	0.8455 (11)	0.025 (4)*	

### Atomic displacement parameters (Å^2^)

**Table:**

	*U*^11^	*U*^22^	*U*^33^	*U*^12^	*U*^13^	*U*^23^
O1	0.0173 (4)	0.0155 (4)	0.0111 (4)	0.0054 (3)	0.0025 (3)	0.0026 (3)
O2	0.0293 (5)	0.0177 (5)	0.0151 (4)	0.0085 (4)	0.0036 (4)	0.0065 (4)
N1	0.0166 (5)	0.0113 (5)	0.0121 (5)	0.0036 (4)	0.0030 (4)	0.0024 (4)
N2	0.0122 (5)	0.0139 (5)	0.0144 (5)	0.0043 (4)	0.0011 (4)	0.0002 (4)
C1	0.0115 (5)	0.0150 (6)	0.0134 (6)	0.0044 (4)	0.0007 (4)	0.0023 (5)
C2	0.0094 (5)	0.0141 (6)	0.0122 (6)	0.0037 (4)	0.0010 (4)	0.0021 (4)
C3	0.0068 (5)	0.0139 (6)	0.0124 (6)	0.0024 (4)	0.0008 (4)	0.0023 (4)
C4	0.0080 (5)	0.0141 (6)	0.0131 (6)	0.0035 (4)	0.0009 (4)	0.0013 (4)
C5	0.0122 (5)	0.0154 (6)	0.0161 (6)	0.0038 (4)	0.0016 (4)	0.0023 (5)
C6	0.0128 (5)	0.0131 (6)	0.0224 (7)	0.0039 (4)	0.0022 (5)	0.0016 (5)
C7	0.0114 (5)	0.0164 (6)	0.0190 (6)	0.0047 (5)	0.0006 (5)	−0.0043 (5)
C8	0.0131 (5)	0.0210 (6)	0.0130 (6)	0.0063 (5)	0.0017 (4)	0.0006 (5)
C9	0.0086 (5)	0.0147 (6)	0.0153 (6)	0.0034 (4)	0.0009 (4)	0.0026 (5)
C10	0.0102 (5)	0.0124 (5)	0.0159 (6)	0.0041 (4)	0.0005 (4)	0.0039 (4)
C11	0.0192 (6)	0.0149 (6)	0.0161 (6)	0.0072 (5)	0.0017 (5)	−0.0002 (5)
C12	0.0180 (6)	0.0146 (6)	0.0113 (6)	0.0052 (5)	0.0032 (5)	−0.0005 (4)
C13	0.0243 (6)	0.0133 (6)	0.0139 (6)	0.0051 (5)	0.0056 (5)	0.0020 (5)
C14	0.0191 (6)	0.0158 (6)	0.0164 (6)	−0.0019 (5)	0.0048 (5)	0.0009 (5)
C15	0.0186 (6)	0.0206 (6)	0.0137 (6)	0.0030 (5)	0.0015 (5)	−0.0002 (5)
C16	0.0238 (6)	0.0174 (6)	0.0152 (6)	0.0039 (5)	0.0011 (5)	0.0048 (5)
C17	0.0186 (6)	0.0144 (6)	0.0159 (6)	−0.0001 (5)	0.0026 (5)	0.0025 (5)
C18	0.0208 (7)	0.0342 (8)	0.0234 (7)	0.0023 (6)	−0.0030 (6)	0.0048 (6)
C19	0.0173 (6)	0.0133 (6)	0.0133 (6)	0.0040 (5)	0.0032 (4)	0.0040 (4)
C20	0.0163 (6)	0.0118 (5)	0.0143 (6)	0.0013 (4)	0.0039 (5)	0.0050 (4)
C21	0.0172 (6)	0.0171 (6)	0.0161 (6)	0.0047 (5)	0.0024 (5)	0.0058 (5)
C22	0.0223 (6)	0.0194 (6)	0.0195 (7)	0.0079 (5)	0.0068 (5)	0.0047 (5)
C23	0.0267 (7)	0.0169 (6)	0.0143 (6)	0.0037 (5)	0.0043 (5)	0.0034 (5)
C24	0.0207 (6)	0.0198 (6)	0.0161 (6)	0.0033 (5)	−0.0007 (5)	0.0057 (5)
C25	0.0166 (6)	0.0179 (6)	0.0186 (6)	0.0045 (5)	0.0037 (5)	0.0063 (5)
C26	0.0400 (9)	0.0340 (8)	0.0169 (7)	0.0124 (7)	0.0050 (6)	0.0001 (6)

### Geometric parameters (Å, °)

**Table:**

O1—C9	1.3743 (15)	C13—H13	0.9300
O1—C1	1.3904 (15)	C14—C15	1.3949 (19)
O2—C1	1.2180 (16)	C14—H14	0.9300
N1—C3	1.3403 (16)	C15—C16	1.3959 (19)
N1—C19	1.4669 (15)	C15—C18	1.5119 (18)
N1—H1	0.883 (19)	C16—C17	1.3905 (18)
N2—C10	1.2833 (16)	C16—H16	0.9300
N2—C11	1.4682 (15)	C17—H17	0.9300
C1—C2	1.4378 (17)	C18—H18A	0.9600
C2—C3	1.4133 (16)	C18—H18B	0.9600
C2—C10	1.4579 (16)	C18—H18C	0.9600
C3—C4	1.4681 (16)	C19—C20	1.5110 (17)
C4—C9	1.4044 (17)	C19—H19A	0.9700
C4—C5	1.4129 (17)	C19—H19B	0.9700
C5—C6	1.3832 (18)	C20—C25	1.3934 (18)
C5—H5	0.9300	C20—C21	1.4008 (18)
C6—C7	1.3945 (19)	C21—C22	1.3877 (19)
C6—H6	0.9300	C21—H21	0.9300
C7—C8	1.3835 (18)	C22—C23	1.4011 (19)
C7—H7	0.9300	C22—H22	0.9300
C8—C9	1.3911 (17)	C23—C24	1.393 (2)
C8—H8	0.9300	C23—C26	1.5116 (19)
C10—H10	0.9300	C24—C25	1.3945 (19)
C11—C12	1.5196 (17)	C24—H24	0.9300
C11—H11A	0.9700	C25—H25	0.9300
C11—H11B	0.9700	C26—H26A	0.9600
C12—C13	1.3955 (18)	C26—H26B	0.9600
C12—C17	1.3956 (18)	C26—H26C	0.9600
C13—C14	1.3928 (19)		
			
C9—O1—C1	121.68 (10)	C13—C14—H14	119.3
C3—N1—C19	131.64 (11)	C15—C14—H14	119.3
C3—N1—H1	111.9 (12)	C14—C15—C16	117.75 (12)
C19—N1—H1	116.0 (12)	C14—C15—C18	120.63 (12)
C10—N2—C11	118.27 (11)	C16—C15—C18	121.62 (12)
O2—C1—O1	114.96 (11)	C17—C16—C15	121.08 (12)
O2—C1—C2	127.14 (11)	C17—C16—H16	119.5
O1—C1—C2	117.90 (10)	C15—C16—H16	119.5
C3—C2—C1	121.68 (11)	C16—C17—C12	120.92 (12)
C3—C2—C10	123.54 (11)	C16—C17—H17	119.5
C1—C2—C10	114.72 (11)	C12—C17—H17	119.5
N1—C3—C2	117.25 (11)	C15—C18—H18A	109.5
N1—C3—C4	124.45 (11)	C15—C18—H18B	109.5
C2—C3—C4	118.30 (11)	H18A—C18—H18B	109.5
C9—C4—C5	116.27 (11)	C15—C18—H18C	109.5
C9—C4—C3	117.57 (11)	H18A—C18—H18C	109.5
C5—C4—C3	126.15 (11)	H18B—C18—H18C	109.5
C6—C5—C4	121.64 (12)	N1—C19—C20	108.29 (10)
C6—C5—H5	119.2	N1—C19—H19A	110.0
C4—C5—H5	119.2	C20—C19—H19A	110.0
C5—C6—C7	120.29 (12)	N1—C19—H19B	110.0
C5—C6—H6	119.9	C20—C19—H19B	110.0
C7—C6—H6	119.9	H19A—C19—H19B	108.4
C8—C7—C6	119.77 (11)	C25—C20—C21	118.50 (12)
C8—C7—H7	120.1	C25—C20—C19	121.85 (11)
C6—C7—H7	120.1	C21—C20—C19	119.65 (11)
C7—C8—C9	119.53 (12)	C22—C21—C20	120.64 (12)
C7—C8—H8	120.2	C22—C21—H21	119.7
C9—C8—H8	120.2	C20—C21—H21	119.7
O1—C9—C8	114.75 (11)	C21—C22—C23	121.13 (12)
O1—C9—C4	122.75 (11)	C21—C22—H22	119.4
C8—C9—C4	122.50 (12)	C23—C22—H22	119.4
N2—C10—C2	123.26 (11)	C24—C23—C22	117.93 (12)
N2—C10—H10	118.4	C24—C23—C26	121.63 (13)
C2—C10—H10	118.4	C22—C23—C26	120.44 (13)
N2—C11—C12	108.96 (10)	C23—C24—C25	121.24 (12)
N2—C11—H11A	109.9	C23—C24—H24	119.4
C12—C11—H11A	109.9	C25—C24—H24	119.4
N2—C11—H11B	109.9	C20—C25—C24	120.57 (12)
C12—C11—H11B	109.9	C20—C25—H25	119.7
H11A—C11—H11B	108.3	C24—C25—H25	119.7
C13—C12—C17	118.25 (12)	C23—C26—H26A	109.5
C13—C12—C11	121.18 (11)	C23—C26—H26B	109.5
C17—C12—C11	120.38 (11)	H26A—C26—H26B	109.5
C14—C13—C12	120.52 (12)	C23—C26—H26C	109.5
C14—C13—H13	119.7	H26A—C26—H26C	109.5
C12—C13—H13	119.7	H26B—C26—H26C	109.5
C13—C14—C15	121.40 (12)		
